# Changes in lifestyle behaviors during the COVID-19 pandemic in children and adolescents with congenital heart disease

**DOI:** 10.1590/1984-0462/2023/41/2022023

**Published:** 2023-03-13

**Authors:** Michele Honicky, Silvia Meyer Cardoso, Luiz Rodrigo Augustemak de Lima, Juliana Nicolodi Souza, Francilene Gracieli Kunradi Vieira, Isabela de Carlos Back, Yara Maria Franco Moreno

**Affiliations:** a Universidade Federal de Santa Catarina, Florianópolis, SC, Brazil; b Universidade Federal de Alagoas, Maceió, Alagoas, Brazil

**Keywords:** COVID-19, Dietary pattern, Sleep, Physical activity, Sedentary behavior, Congenital heart disease, COVID-19, Padrão alimentar, Sono, Atividade física, Comportamento sedentário, Cardiopatia congênita

## Abstract

**Objective:**

To describe the changes in lifestyle behaviors during the COVID-19 pandemic in children and adolescents with congenital heart disease and to investigate the association of congenital heart disease complexity with lifestyle behavior changes.

**Methods:**

Cross-sectional study with 127 children and adolescents with congenital heart disease, who underwent cardiac procedure (mean postoperative time: 10.11±3.13 years), conducted between December 2020 and January 2021. Lifestyle behaviors, such as dietary intake, physical activity, sedentary behavior, and sleep, were assessed through telephone interview based on validated questionnaires. Dietary patterns were identified using principal component analysis. Frequency of general and specific combinations of healthy and unhealthy lifestyle behavior changes was evaluated. Multinomial logistic regressions were used to test the association between congenital heart disease complexity and changes in lifestyle behavior.

**Results:**

The main lifestyle behaviors acquired during pandemic were: 83.5% decreased physical activity; 37.0% increased sedentary behavior; 26.0% slept more than usual; and 23.6% adopted a less-healthy dietary pattern. Almost half of the participants (41.8%) had at least one unhealthy change in lifestyle behavior. Complex congenital heart diseases were associated with increased sedentary behavior (OR 3.49, 95%CI 1.23–9.90).

**Conclusions:**

Children and adolescents with congenital heart disease had unhealthy lifestyle behavior during the pandemic, mainly in the form of reduced physical activity and increased sedentary behavior.

## Resumo

**Objetivo:**

Descrever as mudanças nos estilos de vida durante a pandemia em crianças e adolescentes com cardiopatia congênita e investigar a associação da complexidade da cardiopatia congênita com as mudanças de estilo de vida.

**Métodos:**

Estudo transversal com 127 crianças e adolescentes com cardiopatia congênita, que realizaram procedimento cardíaco (tempo médio de pós-operatório: 10,11 (3,13) anos), realizado entre dezembro de 2020 e janeiro de 2021. O estilo de vida (alimentação, atividade física, comportamento sedentário e sono) foi avaliado por entrevista telefônica, com base em questionários validados. Padrões alimentares foram identificados por meio da análise de componentes principais. Frequência de combinações gerais e específicas de mudanças de estilo de vida saudável e não saudável foram avaliadas. Regressões logísticas multinominais foram utilizadas para testar associações.

**Resultados:**

Os principais comportamentos de estilo de vida adquiridos durante a pandemia foram: 83,5% reduziram a atividade física, 37,0% aumentaram o comportamento sedentário, 26,0% dormiram mais e 23,6% mudaram para um padrão alimentar menos saudável. Quase metade (41,8%) dos participantes teve pelo menos uma mudança não saudável no estilo de vida. Cardiopatias congênitas complexas foram associadas ao aumento do comportamento sedentário durante a pandemia (*odds ratio* 3,49, IC95% 1,23–9,90).

**Conclusões:**

Crianças e adolescentes com cardiopatia congênita apresentaram estilo de vida não saudável durante a pandemia, principalmente na forma de redução da atividade física e aumento do comportamento sedentário.

## INTRODUCTION

COVID-19 infection may also be associated with long-term cardiac complications.^1^ This fact is particularly important in congenital heart disease (CHD) patients since they are also known to be at higher risk of secondary cardiovascular disease (CVD) in early adulthood.^2^ Lifestyle behavior (LB) plays an important role in the development of CVD,^3^ especially in patients with CHD. In Brazil, the preventive measures adopted during the pandemic, particularly at the beginning in February 2020, provoked sudden LB changes^4-6^ that may be a crucial point in the cardiovascular health of children and adolescents with CHD, as they can exacerbate unhealthy LBs. Previous studies found that children and adolescents with CHD already had unhealthy LB before the pandemic, such as unhealthy diet, physical inactivity and sedentary behavior (SB).^7^ Moreover, some studies with healthy children described that the COVID-19 home confinement could be associated with LB changes, both in healthy and unhealthy patterns.^8-10^

However, few studies have examined the LB changes during the pandemic in children and adolescents with CHD. A cross-sectional study with German children and adolescents with CHD found a decrease in physical activity (PA) level during the pandemic, compared to the period before the pandemic.^11^ Thus, LB changes (i.e., dietary patterns [DPs], PA, SB and sleep) during the pandemic remain unclear in children and adolescents with CHD. Besides, there is a lack of studies investigating whether the complexity of CHD influences LB changes during the pandemic. Identifying the effects of the COVID-19 pandemic may help to develop healthy lifestyle promotion strategies for children and adolescents with CHD during periods of home confinement, as well as cardiovascular health promotion strategies post-COVID-19 pandemic. This study aimed to describe the changes in LBs during the pandemic in children and adolescents with CHD and to investigate the association of CHD complexity with LB changes.

## METHOD

**Figure 1.**
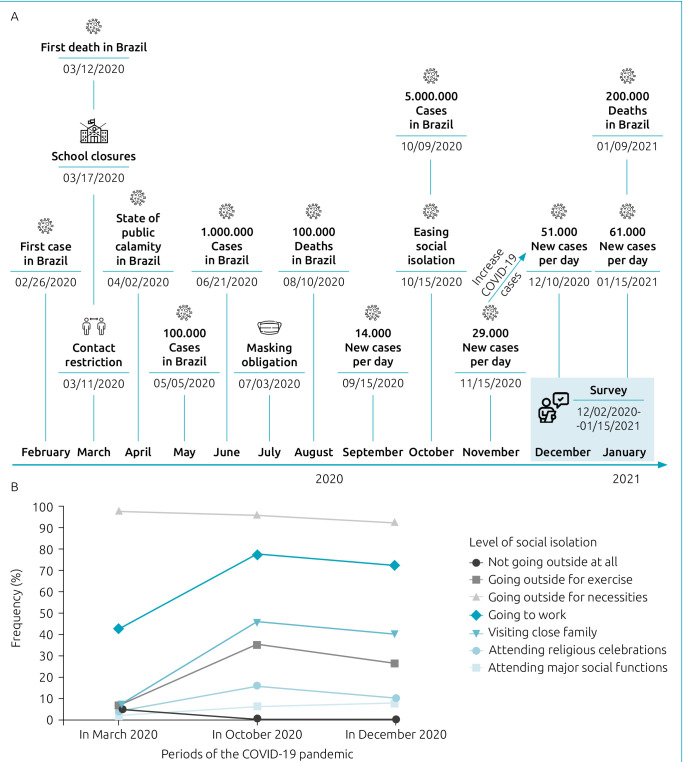
Timeline of events during the COVID-19 pandemic in Brazil and the level of social isolation in children and adolescents with congenital heart disease and their families.

Cross-sectional study derives data from, which includes children and adolescents with CHD aged 5-18 years, who underwent surgery or interventional catheterization for CHD, and postoperative time over 6 months. For the present study, data were collected through telephone interview, between December 2, 2020 and January 15, 2021, which was a period of self-isolation and increasing cases of COVID-19 diagnoses in Brazil. Figure 1A illustrates the timeline of events and survey context during the COVID-19 pandemic in Brazil. The present study recruited children and adolescents with CHD, who underwent surgery or interventional catheterization for CHD from the baseline.^12^ Inclusion criterion was: age between 5 and 18 years. Exclusion criteria were: the presence of genetic syndromes and the presence of chronic or acute inflammatory disease. This study was approved by the Ethics Committee for and complied with the 1964 Helsinki Declaration and its later amendments. Written informed consent was obtained from the parents/legal guardians of all the participants enrolled in the study.

A) Timeline of events and survey context during the COVID-19 pandemic in Brazil*

B) Level of social isolation in children and adolescents with CHD and their families.

*Data from World Health Organization (https://covid19.who.int/region/amro/country/br) and Official Diary of the Union (https://www.in.gov.br/web/guest/servicos/diario-oficial-da-uniao). The timeline of events during the COVID-19 pandemic is an overview of Brazil, because each region of the country had taken different measures of social isolation and closing of schools. In brief, in March we had the beginning of the pandemic, in October, the decree publication on easing COVID-19 lockdowns and restrictions, and in December/January, the period of data collection of the study and increase in the cases of COVID-19. The icons shown in this figure have been designed using freely available resources from Flaticon.com.

Sociodemographic and clinical characteristics were recorded, including age, sex, household income, cardiac procedure, postoperative time and complexity of CHD,^13^ according to the CHD diagnoses (complex, moderate and mild lesions), and classified as simple/moderate or complex.

COVID-19 questionnaire consisted of questions relating to the impact of the pandemic on LBs, specifically dietary intake, PA, SB and sleep, and was adapted based on previous studies on LB changes during the pandemic in children and adolescents.^5,8,9,14^ The structured questionnaire was applied through telephone with an average call duration of 40 minutes. It was applied to the parents/legal guardians and children/adolescents together, in which the children and adolescents helped with the answers. All interviewers received prior training to perform the interview in order to standardize data collection and avoid errors and biases. COVID-19 questionnaire was divided into six sections:

COVID-19 diagnosis and level of isolation: Test positive for severe acute respiratory syndrome coronavirus 2 (SARS-CoV-2) in either participant or family member, symptoms, and severity of symptoms were collected. Level of social isolation was also assessed in three periods of the pandemic: March 2020, October 2020 and December 2020/January 2021. Participants also reported on their routine pediatric cardiologist follow-up during the pandemic.

Financial issues: Reported financial concern and food insecurity during past month were evaluated. Also, the receipt of government assistance during the pandemic was assessed.

Dietary intake: Frequency of 10 food groups intake were obtained: beans/chickpeas/lentils, fried pastries, vegetables, sweets, fruits, milk, meat, eggs, soft drink and ultra-processed foods. In addition, changes in eating habits during the pandemic were also assessed.

PA: PA questions were based on the International Physical Activity Questionnaire (IPAQ), which assesses walking, as well as, moderate and vigorous PA in children and adolescents. Active was defined as ≥300 minutes/week of PA.^15^ PA was categorized into: active before and during the pandemic, active during the pandemic, inactive before and during the pandemic and inactive during the pandemic. Additionally, PA changes compared to the period before the pandemic were categorized as decreased, increased and constant.

SB: Sedentary time was assessed by the sum of hours spent on computers, smartphone and television per day. SB was considered at >2 hours/day^16^ and was categorized into: SB before and during the pandemic, SB during the pandemic, no SB before and during the pandemic and no SB during the pandemic, and also categorized in increased and constant, compared to the period before the pandemic.

Sleep: Sleep duration and quality of sleep during the pandemic were evaluated compared to before the pandemic, and categorized in: better, similar and worse. Also, changes in hours of sleep per night were assessed and categorized into: unchanged, more than usual and less than usual.

Participant’s general characteristics were expressed as means (standard deviation [SD]) or medians (interquartile range [IQR]) for continuous variables and as frequency and percentages for categorical variables. To compare differences in eating habits and sleep before and during the pandemic, the chi-square or Fisher’s exact test were applied. DPs were derived from principal component analysis (PCA) based on the 10 food groups. Input variable was the frequency of food intake categorized in <5 days/week and ≥5 days/week, which uses a tetrachoric correlation matrix from binary data. Factor loadings >0.30 were considered representative of DP. Subsequently, DPs were named according to the food groups in each DP. After extracting the DP, individual scores of each were obtained and divided into: low adherence to DP (<75^(th)^ percentile) and high adherence to DP (≥75^(th)^ percentile). Next, DP changes compared to before the pandemic was categorized as more-healthy and less-healthy according to the change from low adherence to “unhealthy” or “healthy” DP to high adherence to “unhealthy” or “healthy” DP, and vice versa. 

To facilitate interpretations, each change in LBs during the pandemic were categorized into two groups based on healthy and unhealthy LB. Changes in healthy LB were defined as: healthy DP (i.e., change to high adherence to healthy DP and change to low adherence to unhealthy DP); healthy PA (i.e., increased PA); healthy SB (i.e., reduced SB); and healthy sleep (increased sleep duration). Conversely, changes in unhealthy LB were defined as: unhealthy DP (i.e., change to high adherence to unhealthy DP and change to low adherence to healthy DP); unhealthy PA (i.e., reduced PA); unhealthy SB (i.e., increased SB); and unhealthy sleep (i.e., reduced sleep duration). Thereafter, the frequency of general (i.e., all four, three out of four, two out of four, one out of four, none) and specific (i.e., DP, SB, sleep and other specific combinations) combinations of LB changes during the pandemic were assessed.

Multinomial logistic regression model was used to test the association of CHD complexity (exposure) with changes in LB (outcomes). Subsequently, multinomial logistic regressions were adjusted for potential confounding: age, sex and household income. The results were expressed as odds ratio (OR) and respective 95% confidence interval (95%CI).

All statistical tests were conducted using the Statistical Package for Social Sciences SPSS version 23.0 (IBM SPSS Inc.), except for the DPs that were performed by Stata version 13.0 (Stata Corporation). p-values <0.05 were considered statistically significant.

## RESULTS

A total of 127 children and adolescents with CHD were included in the present study. The majority were female (55.9%), and the median age was 12.1 years (10.1–15.0). Only 2.4% (n=3) of participants tested positive for SARS-CoV-2 and all had very mild symptoms. The main symptoms reported were: loss of taste, diarrhea, loss of smell, fatigue, sore throat and fever. Among the participants, 23.6% had parents, siblings or other residents of the same home that tested positive for SARS-CoV-2. Regarding routine pediatric cardiologist follow-up during the pandemic, 40.9% did not consult at all and the main reasons were the following: scheduling difficulties (14.2%); the patient was asymptomatic (8.5%); fear of the COVID-19 infection (7.9%); postponement of routine follow-up until next year (6.3%); and cancellation of appointment due to largely increased COVID-19 cases (3.9%). Regarding the cardiac procedure, 82.7% underwent cardiac surgery and 17.3% underwent cardiac catheterization. The mean postoperative time was 10.1±3.1 years and 77.2% had simple/moderate CHD complexity. Of all the 127 participants’ parents/guardians, 45.7% reported financial concern, 19.7% reported food insecurity, and 46.5% received government assistance during the pandemic.

The majority of participants (94.5%) followed the rules of social isolation from March 2020 to January 2021. Figure 1B describes the levels of social isolation during the COVID-19 pandemic.

**Figure 2.**
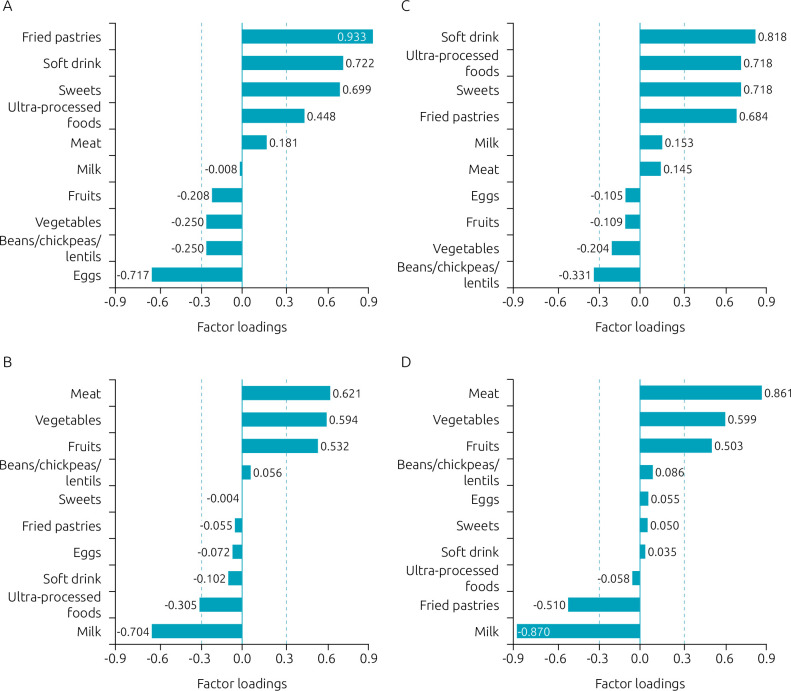
Dietary patterns and factor loadings of food groups before and during the pandemic in children and adolescents with congenital heart disease.

Two DPs were identified before and during the pandemic (DP 1 is unhealthy and DP 2 is healthy), which explained 29.0% and 29.3% of the total variance, respectively. Before the pandemic, DP 1 was characterized by “unhealthy”, with high intake of fried pastries, soft drinks, sweets, and ultra-processed foods and low intake of eggs (Figure 2A), and DP 2 was represented by “healthy”, with high intake of meat, vegetables, fruits, and low intake of milk and ultra-processed foods (Figure 2B). During the pandemic, DP 1 was characterized by “unhealthy”, with high intake of soft drinks, ultra-processed foods, sweets, fried pastries and low intake of beans/chickpeas/lentils (Figure 2C), and DP 2 was represented by “healthy”, with high intake of meat, vegetables, fruits and low intake of milk and fried pastries (Figure 2D). According to the change in DPs during the pandemic, 23.6% of the participants shifted to a less-healthy DP, and 16.5% shifted to a more-healthy DP. 

A) Unhealthy dietary patterns before the pandemic; B) Healthy dietary patterns before the pandemic; C) Unhealthy dietary patterns during the pandemic; D) Healthy dietary patterns during the pandemic.

In addition, significant increases were observed before pandemic vs during pandemic in the frequency of the behaviors as follow: having breakfast (every day: 67.7% vs 74.0%; 1−5 days/week 12.5% vs 8.6%; rarely 7.1% vs 7.1%; no: 16.6% vs 10.2%; p<0.001); meals had together with the family (every day: 88.2% vs 91.3%; 1−5 days/week: 11.8% vs 4%; rarely: 3.1% vs 3.1%; no: 0% vs 1.6%; p<0.001); cooking at home (<5 days/week: 85.8% vs 92.1%; 1−4 days/week: 11.9% vs 7.9%; no: 2.4% vs 0%; p<0.001); and watching TV while eating (every day: 16.5% vs 24.4%; 1−5 days/week: 23.7% vs 30%; rarely: 21.3% vs 14.2%; no: 38.6% vs 31.5%; p<0.001). Significant decreases were observed before pandemic vs during pandemic in the frequency of the following behaviors: consumption at fast-food restaurants (1−5 days/week: 32.3% vs 18.9%; rarely: 45.7% vs 7.9%; no: 22.0% vs 73.2%; p<0.001); and use of apps to order food (<3 days/week: 22% vs 2.6%; 1−2 days/week: 53.6% vs 28.3%; rarely: 0% vs 39.4%; no: 24.4% vs 29.9%; p<0.001).

**Figure 3.**
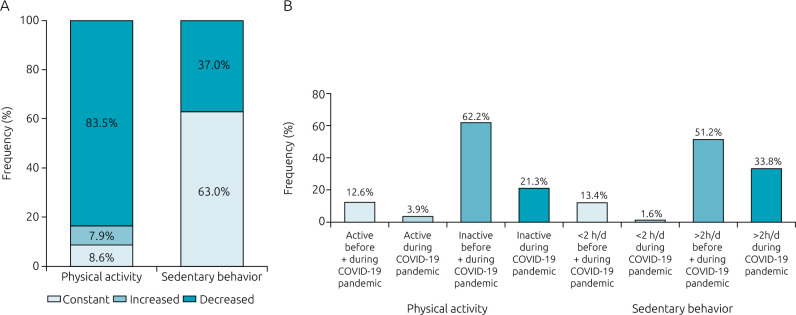
Physical activity and sedentary behavior in children and adolescents with congenital heart disease during pandemic.

During the pandemic, 83.5% of the participants reported decreased PA levels, of which, 62.2% were inactive before and during the pandemic and 21.3% were inactive during the pandemic. SB was increased by 37.0% in the participants, 51.2% had ≥2 hours/day of SB before and during the pandemic and 33.8% had ≥2 hours of SB during the pandemic (Figure 3). Moreover, the screen time for school activities was 90 minutes/day during the pandemic. Regarding school activities, 33.0% of participants received only printed/textbook school activities and 2.4% returned to school in person to receive assistance with school activities.

A) Frequency of change in physical activity and sedentary behavior during the pandemic; B) Frequency of physical activity and sedentary behavior before and during the pandemic. Active was defined as ≥300 minutes/week of physical activity.^15^ Sedentary behavior was considered when >2 hours/day.16 h/d: hours/day

During the pandemic, the reported sleep duration increased by 26.0% in participants, still, the quality of sleep worsened by 26.8%.

**Figure 4.**
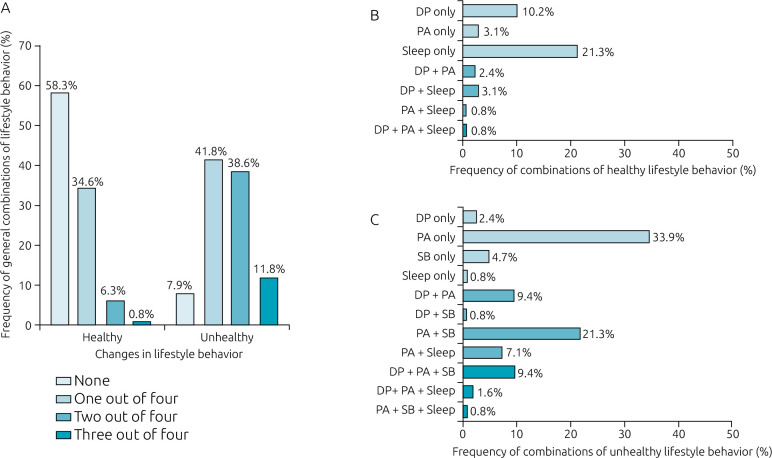
Proportion of participants with healthy and unhealthy lifestyle behavior changes and combinations of healthy and unhealthy lifestyle behavior changes during the pandemic in children and adolescents with congenital heart disease.

Among the 127 participants, 34.6% had one of four healthy behavior changes, and 58.3% had no healthy behavior change during the pandemic (Figure 4A and 4B). Of the 34.6%, sleeping more than usual was the most prevalent behavior (21.3%). As for the unhealthy changes, 41.8% had one of four unhealthy behavior changes and 11.8% had three of four. Of the 41.8% that had one of four unhealthy changes, PA decrease was the most prevalent (33.9%). Moreover, the unhealthy specific combination of DP, PA and SB was identified in 9.4% of the participants (Figure 4C).

A) Frequency of general combinations of lifestyle behaviors; B) Frequency of specific combinations of healthy lifestyle behaviors; C) Frequency of specific combinations of unhealthy lifestyle behaviors. DP: dietary pattern; PA: physical activity; SB: sedentary behavior.

In multivariable-adjusted multinomial logistic regressions, complex CHD were associated with increased SB vs constant SB during the pandemic, compared with simple and moderate CHD. CHD complexity was not associated with other LB changes during the pandemic (Table 1).

**Table 1.** Association of congenital heart disease complexity and changes in lifestyle behaviors in children and adolescents with congenital heart disease.

Outcomes	CHD complexity	
Changes in lifestyle behaviors	Simple/moderate CHD	Complex CHD
	OR (95%CI); p-value*	
Change in DPs		
Healthy vs unchanged	1	3.00 (1.00–8.97); 0.049
Unhealthy vs unchanged	1	1.54 (0.48–4.90); 0.468
Change in physical activity		
Increased vs constant	1	0.65 (0.08–5.04); 0.677
Decreased vs constant	1	1.00 (0.22–4.53); 0.997
Change in sedentary behavior		
Increased vs constant	1	3.49 (1.23–9.90); 0.019
Decreased vs constant	-	-
Change in sleep duration		
More than usual vs constant	1	0.41 (0.15–1.11); 0.080
Less than usual vs constant	1	0.21 (0.05–1.00); 0.051
Change in sleep quality		
Better vs unchanged	1	0.33 (0.11–1.00); 0.049
Worse vs unchanged	1	0.51 (0.17–1.52); 0.226

CHD: congenital heart disease; OR: odds ratio; CI: confidence interval; DPs: dietary patterns. *Adjusted for sex (male versus female), age (years), income (<1 wage versus >1wage).

## DISCUSSION

In this cross-sectional study, the main unhealthy LBs changes were physical inactivity and SB. Almost half of the children and adolescents with CHD had at least one unhealthy change in LB during the pandemic. These results reinforce the need to promote effective healthy LBs during and after the COVID-19 pandemic in this population.

One of the behavioral changes during the pandemic in children and adolescents with CHD was dietary intake, some healthy and others unhealthy. In line with previous studies that showed healthy^10,14^ and unhealthy^5,17^ eating habits during the pandemic in the pediatric population, Massin^7^ reported that before the pandemic, children with CHD had a high intake of sugary drinks and foods high in fats and low intake of fruit and vegetables. Our findings suggest that home confinement exacerbated these unhealthy eating habits. In the present study, a shift of a less-healthy DP was found in 23.6% of participants during the pandemic, they acquired other unhealthy eating habits, such as watching TV while eating. This behavior is associated with high ultra-processed foods intake.^18^ Although some participants increased unhealthy eating habits, there are also notable changes in healthy eating habit reported during the pandemic in this study. For example, frequency increase of having breakfast, having meals together with the family and cooking at home, which are positively associated with overall diet quality in healthy children.^19^ Moreover, our finding on increased cooking at home is similar to the results of a study in Brazilian adults^4^ and Canadian families.^8^ However, it is worth noting that confinement may have increased the availability of time to cook at home, but this may not necessarily represent a healthier diet. Furthermore, a previous study with children and adolescents with CHD found that approximately 36% went to fast-food restaurants once a month before the pandemic.^7^ In the present study, as expected, home confinement resulted in a decreased in the frequency of consumption at fast-food restaurants and in the use of app to order food. It is also noteworthy that the present study found that healthy DP during the pandemic was composed by low intake of fried pastries. In contrast, adolescents from South America and Europe showed an increase of fried foods intake during the pandemic.^20^

Before the pandemic, previous studies already described high prevalence of physical inactivity in CHD patients.^7,12^ Our findings suggest that self-isolation measures have presented new challenges in practicing moderate to vigorous intensity PA, resulting in a decrease of 83.5% of PA levels, whereas, only 3.9% were active during the pandemic. These results are in line with a German study that found a reduction of almost a quarter in daily step count in children and adolescents with CHD during the pandemic.^11^ Similar results were described in healthy children^6,9,10,21^ and in children with obesity.^17^ Moreover, self-isolation resulted in increased leisure time and SBs such as prolonged sitting and excess of screen time in healthy children and adolescents around the world.^6,9,10^ Results in the same direction were observed in the present study. Additionally, it was observed that the participants used 90 minutes/day of school activities on screen time. However, a study with American parents suggested that remote education is not the greatest contributor to SB during the pandemic, as children spend less time doing school-related video calls than sedentary leisure behaviors such as watching television/videos/movies.^21^ Furthermore, a study with children and adolescents found that the perceived capability of parents to restrict their children’s screen time is one of the main factors of SB during the pandemic.^22^ This suggests the important role of the family in reducing SB, especially inactive leisure, which should be taken into account in the management of these patients by the association between SB and CVD, regardless of PA.^23^ In addition, patients with complex CHD had a higher risk of intensifying SB during the pandemic in the present study, which indicates that this group are advised to pay closer attention to the guidelines in order to promote a healthy LB.

In the present study, the sleep duration increased in 26.0% of the participants during the pandemic. Similar results were described in healthy children.^6,9,14^ A study in Brazil showed that 32% of healthy children increased sleep time^6^ and, a longitudinal study in Italy reported an average growth of one hour.^17^ By contrast, a study from Canada showed that sleep remained the same in young children in middle and high-income families,^8^ which differs from our predominantly low-income population. The school closures may have reflected in children’s sleep changes. Another possible explanation for the growth in sleep time during the pandemic would be due to the lack of establishing hours for bedtime and waking-up.^24^

A combination of LBs should be evaluated to make more effective LB interventions.^25^ The Canadian 24-hour movement guidelines for children and youth reinforces the importance of recommendations regarding LB combinations.^26^ In the present study, 21.3% of participants presented the combination of two unhealthy LBs (i.e., physical inactivity and SB) and 9.4% of participants presented the combination of three unhealthy LBs (i.e., DP, PA and SB), highlighting the importance of interventions in a set of LBs during the pandemic, not just isolated behaviors.

COVID-19 restrictive policies had a predominantly unhealthy effect on PA, SB and DP in children and adolescents with CHD, those changes together can lead to the development of CVD. Previous studies with healthy children and adolescents had already suggested recommendations for promoting healthy lifestyles during the pandemic, such as performing home-based leisure activities with the family, who should support their children to be active, set routines, supervise screen time, curb SB, impose regular bedtime and waking-up times, use social media to connect with friends to minimize distance, and practice mindfulness among others.^9,27^ These recommendations were also imposed on CHD patients to avoid long-term unfavorable cardiovascular outcomes. In addition, encouraging the maintenance of routine care with a pediatric cardiologist and nutritional guidelines for healthy DP are essential components for a healthy LB during and after the pandemic, especially in low-income CHD families, as food insecurity is associated with unhealthy LB and cardiovascular risk.^28^

The present study has some limitations: the cross-sectional design does not allow inferring causality and real changes in LB during the pandemic; self- and parent-reported LB through questionnaire may be underestimated or overestimated due to memory recall bias; food frequency assessment due to difficulty in estimating portions through telephone interviews; data collection during the holiday season. However interviews were conducted a week after the holiday season to avoid bias. Among the strengths: isolated and combined LBs were evaluated.

In conclusion, children and adolescents with CHD had unhealthy lifestyle changes during the pandemic, mainly reduced PA and increased SB. Likewise, patients with complex CHD were associated to higher odds of increase SB. These unhealthy LBs acquired during the pandemic may have an impact on cardiovascular health in the long-term. Thus, our results emphasize the importance of strategies for the CVD prevention in post-COVID-19 pandemic to avoid keeping the unhealthy lifestyle acquired during home confinement, especially in the high-risk group for CVD, such as that with CHD.
